# MADS-box gene family in rice: genome-wide identification, organization and expression profiling during reproductive development and stress

**DOI:** 10.1186/1471-2164-8-242

**Published:** 2007-07-18

**Authors:** Rita Arora, Pinky Agarwal, Swatismita Ray, Ashok Kumar Singh, Vijay Pal Singh, Akhilesh K Tyagi, Sanjay Kapoor

**Affiliations:** 1 Interdisciplinary Centre for Plant Genomics and Department of Plant Molecular Biology, University of Delhi South Campus, Benito Juarez Road, New Delhi 110 021, India; 2 Division of Genetics, Indian Agricultural Research Institute, New Delhi-110 012, India

## Abstract

**Background:**

MADS-box transcription factors, besides being involved in floral organ specification, have also been implicated in several aspects of plant growth and development. In recent years, there have been reports on genomic localization, protein motif structure, phylogenetic relationships, gene structure and expression of the entire MADS-box family in the model plant system, *Arabidopsis*. Though there have been some studies in rice as well, an analysis of the complete MADS-box family along with a comprehensive expression profiling was still awaited after the completion of rice genome sequencing. Furthermore, owing to the role of MADS-box family in flower development, an analysis involving structure, expression and functional aspects of MADS-box genes in rice and *Arabidopsis *was required to understand the role of this gene family in reproductive development.

**Results:**

A genome-wide molecular characterization and microarray-based expression profiling of the genes encoding MADS-box transcription factor family in rice is presented. Using a thorough annotation exercise, 75 MADS-box genes have been identified in rice and categorized into MIKC^c^, MIKC*, Mα, Mβ and Mγ groups based on phylogeny. Chromosomal localization of these genes reveals that 16 MADS-box genes, mostly MIKC^c^-type, are located within the duplicated segments of the rice genome, whereas most of the M-type genes, 20 in all, seem to have resulted from tandem duplications. Nine members belonging to the Mβ group, which was considered absent in monocots, have also been identified. The expression profiles of all the MADS-box genes have been analyzed under 11 temporal stages of panicle and seed development, three abiotic stress conditions, along with three stages of vegetative development. Transcripts for 31 genes accumulate preferentially in the reproductive phase, of which, 12 genes are specifically expressed in seeds, and six genes show expression specific to panicle development. Differential expression of seven genes under stress conditions is also evident. An attempt has been made to gain insight into plausible functions of rice MADS-box genes by collating the expression data of functionally validated genes in rice and *Arabidopsis*.

**Conclusion:**

Only a limited number of MADS genes have been functionally validated in rice. A comprehensive annotation and transcriptome profiling undertaken in this investigation adds to our understanding of the involvement of MADS-box family genes during reproductive development and stress in rice and also provides the basis for selection of candidate genes for functional validation studies.

## Background

The MADS-box family members, identified initially as floral homeotic genes, are one of the most extensively studied transcription factor genes in plants [1-8]. The word MADS finds its origin from the first letters of its founding members, *Mini Chromosome Maintenance 1 *(*MCM1*) of yeast (*Saccharomyces cerevisiae*) [9], *Agamous *(*AG*) of *Arabidopsis *(*Arabidopsis thaliana*) [10], *Deficiens *(*DEF*) of snapdragon (*Antirrhinum majus*) [11] and *Serum Response Factor *(*SRF*) of humans (*Homo sapiens*) [12]. MADS-box transcription factors are characterized by the presence of an approximately 60 amino acids DNA binding domain, known as the MADS-box domain, located in the N-terminal region of the protein. The plant-specific MIKC-type MADS-box proteins include three additional domains followed by the **M**ADS domain, viz. a less-conserved **I**ntervening region of ~30 amino acids, a moderately conserved **K**eratin-like domain of ~70 amino acids mainly involved in heterodimerization, and a highly variable **C**-terminal region of variable length implicated in transcriptional activation and higher-order complex formation [13-15].

The MADS-box family has been divided into two main groups. The type I consists of *ARG80/SRF*-like genes of animals and fungi, also designated as M-type genes in plants, and type II contains *MEF2*-like genes of animals and yeast as well as MIKC-type genes of plants. It is proposed that an ancestral duplication before the divergence of plants and animals gave rise to these groups [16]. The MIKC-type genes are also characterized by the presence of K domain that could have evolved after the divergence of these lineages. The type II genes have been categorized into MIKC^c^- and MIKC*-type based on structural features [17,18]. The MIKC^c ^genes have been further classified into 14 clades based on phylogeny [19,20]. Type I genes have also been categorized into M- and N-type based on the protein motifs identified using the MEME search tool [21] and also as Mα, Mβ, Mγ and Mδ, based on the phylogenetic relationships between MADS-box regions [6]. The Mδ group, however, corresponds to the MIKC* class described in this report and elsewhere [22].

The most striking feature of the MADS-box gene family is the diverse functions taken up by its members in different aspects of plant growth and development. These include flowering time control, meristem identity, floral organ identity, formation of dehiscence zone, fruit ripening, embryo development as well as development of vegetative organs such as root and leaf [23-27]. Genome-wide identification and phylogenetic analyses of MADS-box genes have revealed 107 and 71 (only 65 of these are listed in The Institute for Genomic Research (TIGR) Rice Pseudomolecule release 4 database) genes in *Arabidopsis *and rice, respectively [6,28].

Though a large amount of expression data based on SAGE, microarrays and other high-throughput transcriptome analysis techniques is available in public databases, the studies involving expression of the entire MADS-box family have so far been restricted to northern blot analysis or reverse transcriptase PCR at limited stages of development [6]. Recently, the comparison of expression profiles resulting from a 22 k rice cDNA microarray-based transcriptome analysis of early panicle development in rice was used to implicate three MADS-box genes, *OsMADS1, 14 *and *15*, in panicle branching [29]. The use of high-throughput genome-wide transcriptome analysis provides an insight into changes in the entire transcriptome across a variety of biological conditions. In combination with the whole genome sequence data and comparative expression analysis with genes of known functions, the transcriptomic data can become an initiation point for systematic investigations into structure-function relationships.

With an overall objective to understand regulation of reproductive organ development in *indica *rice, we have initiated a program on microarray-based expression profiling of transcription factors and signal transduction components. Here, we report a comprehensive account of identification and phylogenetic analysis of 75 members of MADS-box gene family in rice and their expression profiling during 11 stages of panicle and seed development along with three abiotic stress conditions and 3 stages of vegetative development. This analysis is based on TIGR Rice Pseudomolecule release 4 and KOME (Knowledge-based *Oryza *Molecular biological Encyclopedia) rice full-length cDNA database. We have identified 10 new members belonging to this gene family besides confirming 65 previously identified genes. Out of 71 previously identified genes by Nam and coworkers [28], six were not found in version 4 of TIGR. Our analysis also suggests the existence of Mβ-type genes in rice, which was earlier thought to be absent in monocots [6]. The results of expression profiling have been discussed in light of phylogenetic relatedness of the genes and their known functions in rice as well as other systems.

## Results

### Identification, organization and structure of MADS-box genes

**Figure 1:**
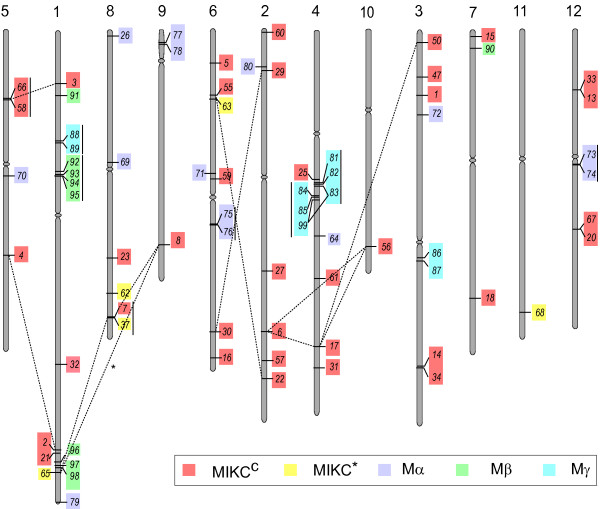
**Chromosomal location of rice MADS-box genes**. 75 MADS-box genes have been mapped on rice chromosomes according to 5' and 3' coordinates mentioned in TIGR. Respective chromosome numbers are written at the top. Genes belonging to five groups have been marked by different colors (MIKC^c^, red; MIKC*, yellow; Mα, purple; Mβ, green and Mγ, blue). Genes lying on duplicated segments of genome have been joined by dashed lines. Tandemly duplicated genes are joined with vertical lines. (* The duplicated segment between chromosome 1 and 9 containing *OsMADS65 *and *8*, respectively, could be detected only in segmental duplication database with 500 kb and not with 100 kb distance allowed between collinear genes in TIGR, for details *see *experimental procedures).

HMM analysis and name search resulted in the identification of 75 MADS-box genes in rice genome. Since, gene names from *OsMADS1 *to *OsMADS58 *representing 34 genes already existed in the literature (though not in continuation), newly identified genes were named from *OsMADS59 *to *OsMADS99 *(*see *Table 1). The individual genes were localized on chromosomes based on the 5' and 3' coordinates for respective gene models in TIGR database (Figure 1). The maximum number of genes (16; 21%) were found to be localized on chromosome 1, whereas, chromosomes 10 and 11 had only one MADS-box gene each. Out of five types of MADS-box genes, the Mγ genes were confined to chromosome 1, 3 and 4, while Mβ genes were present only on chromosome 1. No chromosomal bias was observed in the distribution of MIKC^c^, MIKC* and Mα genes (Figure 1). Analysis of the TIGR rice segmental duplication database revealed 30 MADS-box genes within the duplicated segments of rice chromosomes. Only 16 genes, however, were found to have their counterparts on duplicated segments (Figure 1). Most of the duplicated genes belonged to the MIKC^c ^group. Several MADS-box genes, especially M-type, were also found juxtaposed on chromosomes 1, 4, 5, 6, 8 and 12. Twenty such genes showed significant sequence identity.

**Table 1:** List of 75 MADS-box genes identified in rice and their sequence characteristics (bp, base pair; aa, amino acids; D, Dalton).

**S.No.**	**Name**	**Accession Number**		**ORF (bp)**	**Protein**			**Introns**	**Type**
						
		**TIGR**	**KOME**		**Length (aa)**	**Mol. Wt (d)**	**PI**		
1	OsMADS1	LOC_Os03g11614	AK070981 AK069728	774	257	29.53	7.34	7	MIKC^c^
2	OsMADS2	LOC_Os01g66030	AK070894	630	209	24.16	7.7	6	MIKC^c^
3	*OsMADS3	LOC_Os01g10504	AK108568	864	287	32.12	9.33	8	MIKC^c^
4	OsMADS4	LOC_Os05g34940	AK100233	633	210	24.38	9.1	6	MIKC^c^
5	OsMADS5	LOC_Os06g06750	AK064184	678	225	25.99	7.46	7	MIKC^c^
6	OsMADS6	LOC_Os02g45770	AK069103	753	250	28.44	9.33	7	MIKC^c^
7	OsMADS7/45	LOC_Os08g41950	AK100263	933	310	36.12	10.12	9	MIKC^c^
8	OsMADS8/24	LOC_Os09g32948	AK072867	747	248	28.53	9.21	7	MIKC^c^
9	OsMADS13	LOC_Os12g10540	AK070425	813	270	30.27	9.51	7	MIKC^c^
10	OsMADS14	LOC_Os03g54160	AK121171	762	253	29.21	9.32	7	MIKC^c^
11	OsMADS15	LOC_Os07g01820	AK072683	804	267	30.39	9.49	7	MIKC^c^
12	OsMADS16	LOC_Os06g49840	AK069317	675	224	25.46	8.63	6	MIKC^c^
13	OsMADS17	LOC_Os04g49150	AK070540	765	254	28.80	8.97	7	MIKC^c^
14	OsMADS18/28	LOC_Os07g41370	AK064704	750	249	28.28	9.29	7	MIKC^c^
15	OsMADS20	LOC_Os12g31748	AY250075	522	233	20.44	6.95	5	MIKC^c^
16	OsMADS21	LOC_Os01g66290	AK070958	798	265	29.39	8.62	7	MIKC^c^
17	OsMADS22	LOC_Os02g52340	AK070121	687	228	25.59	5.41	7	MIKC^c^
18	OsMADS23	LOC_Os08g33488	NA	480	159	18.36	9.8	4	MIKC^c^
19	OsMADS25	LOC_Os04g23910	AK102927	684	227	26.14	8.95	7	MIKC^c^
20	OsMADS26	LOC_Os08g02070	AK069122	669	222	25.22	7.45	6	MIKC^c^
21	OsMADS27	LOC_Os02g36924	NA	723	240	27.42	9.48	7	MIKC^c^
22	OsMADS29	LOC_Os02g07430	AK109522	783	260	28.99	6.85	7	MIKC^c^
23	OsMADS30	LOC_Os06g45650	NA	666	221	25.78	7.51	7	MIKC^c^
24	OsMADS31	LOC_Os04g52410	NA	537	178	21.25	10.21	3	MIKC^c^
25	OsMADS32	LOC_Os01g52680	NA	591	196	22.53	8.2	6	MIKC^c^
26	OsMADS33	LOC_Os12g10520	NA	609	202	23.23	9.85	6	MIKC^c^
27	OsMADS34	LOC_Os03g54170	AK100227	720	239	26.88	7.55	7	MIKC^c^
28	OsMADS37	LOC_Os08g41960	NA	612	203	22.56	10.36	3	MIKC*
29	OsMADS47	LOC_Os03g08754	NA	753	250	27.91	6.57	7	MIKC^c^
30	OsMADS50	LOC_Os03g03100	AK104921	351	116	13.28	11.2	6	MIKC^c^
31	OsMADS55	LOC_Os06g11330	AK111859	672	223	24.84	4.74	7	MIKC^c^
32	OsMADS56	LOC_Os10g39130	AK070135	702	233	26.31	10.03	7	MIKC^c^
33	OsMADS57	LOC_Os02g49840	AK108784	726	241	27.24	9.16	7	MIKC^c^
34	OsMADS58	LOC_Os05g11414	AK111723	702	233	25.87	9.28	8	MIKC^c^
35	OsMADS59	LOC_Os06g23950	NA	234	77	85.96	10.29	1	MIKC^c^
36	OsMADS60	LOC_Os02g01360	AK121824	672	223	24.57	8.14	1	MIKC^c^
37	OsMADS61	LOC_Os04g38770	NA	300	99	11.30	10.04	2	MIKC^c^
38	OsMADS62	LOC_Os08g38590	NA	1014	337	36.19	4.44	8	MIKC*
39	OsMADS63	LOC_Os06g11970	AK111776	1083	360	40.07	4.4	10	MIKC*
40	OsMADS64	LOC_Os04g31804	NA	750	249	27.87	6.95	6	Mα
41	OsMADS65	LOC_Os01g69850	AK066160	495	164	18.36	10.28	4	MIKC*
42	OsMADS66	LOC_Os05g11380	NA	303	100	11.04	5.09	1	MIKC^c^
43	OsMADS67	LOC_Os12g31010	ABA98556	162	53	6.36	11.27	0	MIKC^c^
44	OsMADS68	LOC_Os11g43740	NA	1158	385	42.67	6.62	10	MIKC*
45	OsMADS69	LOC_Os08g20460	NA	555	184	20.10	9.29	0	Mα
46	OsMADS70	LOC_Os05g23780	NA	657	218	24.08	8.81	0	Mα
47	OsMADS71	LOC_Os06g22760	NA	717	238	27.03	10.09	0	Mα
48	OsMADS72	LOC_Os03g14850	NA	558	185	20.58	10.05	1	Mα
49	OsMADS73	LOC_Os12g21850	NA	585	194	21.19	11.3	0	Mα
50	OsMADS74	LOC_Os12g21880	NA	360	119	12.83	9.2	1	Mα
51	OsMADS75	LOC_Os06g30810	NA	633	210	22.76	4.79	0	Mα
52	OsMADS76	LOC_Os06g30830	NA	726	241	25.71	9.22	2	Mα
53	OsMADS77	LOC_Os09g02780	NA	537	178	19.30	4.55	0	Mα
54	OsMADS78	LOC_Os09g02830	NA	627	208	22.95	10.79	0	Mα
55	OsMADS79	LOC_Os01g74440	NA	627	208	20.90	10.72	0	Mα
56	OsMADS80	LOC_Os02g06860		861	286	30.76	6.59	0	Mα
57	OsMADS81	LOC_Os04g24790	NA	630	209	23.21	10.08	0	Mγ
58	OsMADS82	LOC_Os04g24800	NA	630	209	23.26	10.01	0	Mγ
59	OsMADS83	LOC_Os04g24810	NA	630	209	23.58	10.63	0	Mγ
60	OsMADS84	LOC_Os04g25870	NA	630	209	23.39	10.2	0	Mγ
61	OsMADS85	LOC_Os04g25920	NA	630	209	23.29	9.02	0	Mγ
62	OsMADS86	LOC_Os03g37670	NA	765	254	28.31	9.03	4	Mγ
63	OsMADS87	LOC_Os03g38610	NA	750	249	27.54	9.93	0	Mγ
64	OsMADS88	LOC_Os01g18420	NA	723	240	26.42	10.69	0	Mγ
65	OsMADS89	LOC_Os01g18440	NA	921	306	33.21	6.67	0	Mγ
66	OsMADS90	LOC_Os07g04170	NA	1473	490	54.43	4.86	4	Mβ
67	OsMADS91	LOC_Os01g11510	NA	1815	604	65.53	4.37	0	Mβ
68	OsMADS92	LOC_Os01g23750	NA	1221	406	42.53	4.38	0	Mβ
69	OsMADS93	LOC_Os01g23760	NA	1224	407	42.73	4.38	0	Mβ
70	OsMADS94	LOC_Os01g23770	NA	972	323	34.46	7.23	2	Mβ
71	OsMADS95	LOC_Os01g23780	NA	837	278	28.86	5.85	2	Mβ
72	OsMADS96	LOC_Os01g67890	NA	1452	483	52.84	6.94	0	Mβ
73	OsMADS97	LOC_Os01g68420	NA	819	272	28.79	4.41	0	Mβ
74	OsMADS98	LOC_Os01g68560	NA	1440	479	51.15	4.3	0	Mβ
75	OsMADS99	LOC_Os04g25930	NA	465	154	17.39	10.58	1	Mγ

*Molecular wt and PI of OsMADS3 has been calculated using Gene Runner

** TIGR Model Represents only K box protein.

Similar to that reported in *Arabidopsis*, distribution of introns in rice MADS-box family genes was also found bimodal with MIKC^c ^and MIKC* genes containing multiple introns and the Mα, Mβ and Mγ genes usually having no or occasionally up to 4 introns (*see *Table 1; [6]). The length of MADS-box proteins varied from 150 to 300 amino acids, with few exceptionally longer or smaller proteins (Table 1). For details on other parameters of nucleic acid and protein sequences, refer to Table 1.

### Evolutionary relationships between rice and Arabidopsis MADS-box family genes

To examine the evolutionary relationships of MADS-box genes in rice (including the 10 new genes identified in this study) and *Arabidopsis*, a tree was constructed using only the conserved MADS-box domain. Five groups, as described by Parenicova and coworkers [6] were identified containing representative genes of both rice and *Arabidopsis*. All the *Arabidopsis *proteins were found to lie in groups similar to those identified previously [6], except AGL47 and AGL82, which instead of forming a basal branch of the Mβ, grouped with Mγ proteins in our analysis as shown in supplementary figure S1 [see Additional file 1]. OsMADS64 grouped separately with AGL33 of *Arabidopsis*, which does not cluster with any of the MADS groups described above [see Additional file 1].

**Figure 2:**
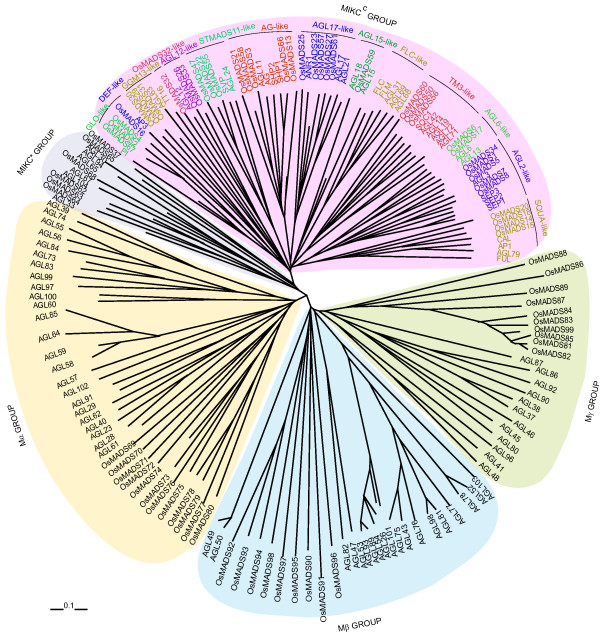
**Phylogenetic analysis of rice and Arabidopsis MADS-box proteins**. Phylogenetic analysis of 173 MADS-box proteins from rice (75) and *Arabidopsis *(98), showing similar groups in both the plant species as given by Parenicova and coworkers [6]. Total of 14 clades formed by MIKC^c^-type genes are also marked. Scale bar represents 0.1 amino acid substitution per site.

A separate phylogenetic tree was also generated from complete protein sequences of all the MADS-box genes in rice and *Arabidopsis *(Figure 2). Of the 75 rice MADS-box genes, 38 grouped with MIKC^c^, six with MIKC*, nine with Mβ, 13 with Mα and 10 grouped with Mγ-type *Arabidopsis *genes. In case of M-type genes, barring *OsMADS90*, *91 *and *96*, all other genes exhibited similar groupings as in the MADS-domain-specific phylogenetic analysis (Figure 2). MIKC^c ^proteins were further divided into 14 clades. Representatives of both rice and *Arabidopsis *could be identified in all the clades except *OsMADS32 *and the *FLC *clade, which are exclusive to rice and *Arabidopsis*, respectively (Figure 2). Phylogenetic trees with bootstrap values, for complete protein sequences as well as coding sequences of rice and *Arabidopsis *MADS-box genes have been shown as supplementary figures S2 and S3, respectively [see Additional files 2 and 3].

### Distribution of conserved motifs

**Figure 3:**
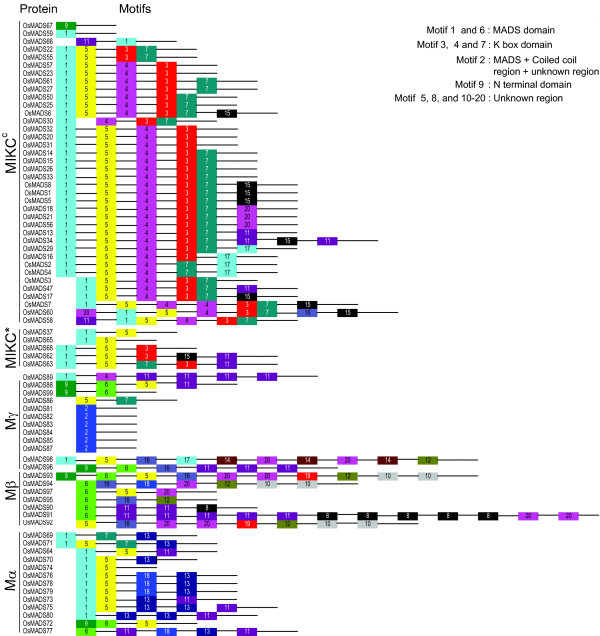
**Distribution of Conserved motifs in rice MADS-box proteins identified using MEME search tool**. Schematic representation of motifs identified in rice MADS-box proteins using MEME motif search tool for each group given separately. Each motif is represented by a number in colored box. Length of box does not correspond to length of motif. Order of the motifs corresponds to position of motifs in individual protein sequence. For detail of motifs refer to supplementary material.

The MEME motif search tool was employed to identify the conserved motifs present in MADS-box proteins (Figure 3). Motifs 1, 6 or 2 specifying the MADS domain were found in all the members of the MADS-box family. All proteins belonging to MIKC^c ^and MIKC* groups had motif 1-type MADS domain. Most Mα proteins also had the motif 1-type MADS domain except OsMADS72 and 77 which contained motif 6. Motif 6 was found to be the most common type of MADS domain in Mβ-type proteins. Distinctively, in case of Mγ proteins a larger MADS domain of 83 amino acids was detected, followed by a coiled coil region and a region of unknown complexity as indicated by Simple Modular Architecture Research Tool (SMART) version 3.4. Together these components formed a larger conserved region of 200 amino acids denoted as motif 2. The conserved motifs 4, 3 and 7, specifying K domain, were found mainly in the MIKC^c ^group members. Of the 38 MIKC^c ^proteins, 31 had all three conserved regions, whereas, motif 7 was absent in case of OsMADS23 and 57; motif 4 in OsMADS22 and 55; and the complete K domain was missing in OsMADS67, 59 and 66. The partial K domain was also detected in some of the non-MIKC^c ^proteins including OsMADS89 and 86 of Mγ, OsMADS68 and 62 of MIKC*, OsMADS63 and OsMADS69 of Mα group.

Certain unknown motifs, numbered 5, 11, 15, 16, 17 and 20, besides the MADS (motif 1) and the K (motifs 3, 4, and 7) domains in MIKC^c ^proteins, were also revealed by MEME motif search. Motif 5 corresponded to the intervening region between the MADS and the K domains, whereas, the rest were found to be distributed in the C-terminal regions. The MADS domain in five M-type proteins, OsMADS88, 99, 96, 93 and 72, was found to be preceded by an N-terminal domain (motif 9). In Mβ proteins, the MADS domain was represented by motif 6 except in OsMADS98 and 92. In addition, other motifs, viz. 5, 8, 10, 12, 14, 16, 17, 19 and 20 were also detected, of which motifs 8, 10, 12, 14 and 19 were exclusive to the Mβ class of proteins. The sequences and lengths of all the motifs are given in supplementary table S1 [see Additional file 4].

### Expression profiling of MADS-box genes during vegetative and reproductive development and stress

**Figure 4:**
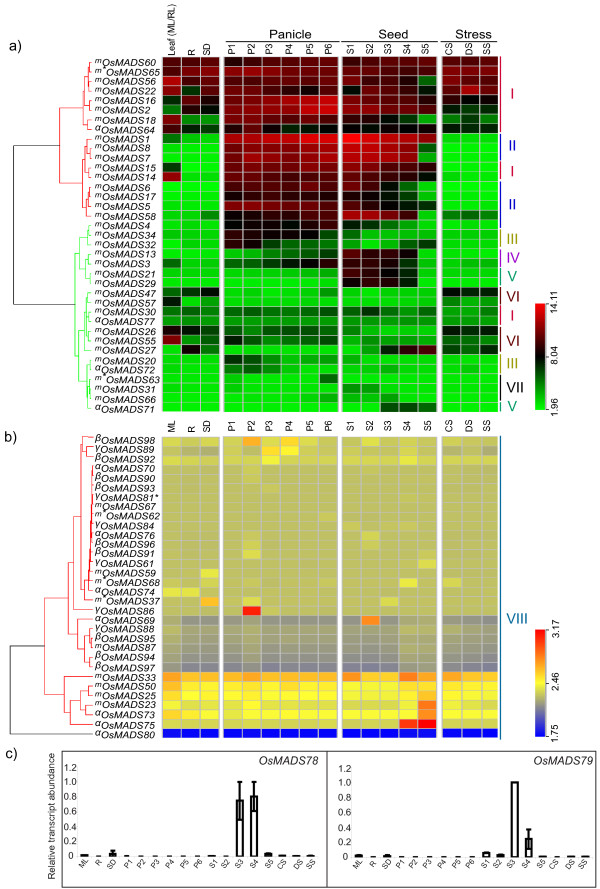
**Expression Analysis of MADS-box genes in rice**. ***a)** Hierarchical cluster display of expression profile for thirty seven MADS-box genes showing high level expression in rice*. (Color bar at the base represents log_2 _expression values, thereby green color representing low level expression, black shows medium level expression and red signifies high level expression). ***b) ****Hierarchical cluster display of expression profile for thirty two MADS-box genes showing low level expression in rice*. (Color bar at the base represents log_2 _expression values, thereby blue color representing low level expression, yellow shows medium level expression and red signifies high level expression). Developmental stages used for expression profiling are mentioned on top of each column. Panicle and seed stages have been listed in the temporal order of development. On the left side of expression map, cluster dendrogram is shown. On the right side, eight groups have been made for the genes showing discrete expression patterns (Figure 5). Symbol * represents accumulative expression profiles of duplicated genes, *OsMADS81*, *82*, *85 *and *99*. Genes belonging to the different groups have been marked by a symbol (m, MIKC^c^; m*, MIKC*; α, Mα; β, Mβ and γ, Mγ). ***c) ****Results of QPCR for OsMADS78 and 79*. Expression profiles of *OsMADS78 *and *79 *using QPCR. X-axis represents the developmental stages as given in Table 2. Y-axis represents relative expression values obtained after normalizing against maximum expression value. Error bars show the standard error for two biological replicates performed.

For expression analysis, the rice panicle and seed development stages were divided into 6 and 5 broad categories, respectively, based on landmark developmental events as described by Itoh and coworkers [30]; information available at oryzabase [31] and our preliminary histochemical analysis (data not shown; Table 2). Seedlings subjected to three stress conditions, viz. desiccation, cold and salt stress, were also included in this analysis. Transcriptome profiling of these stages along with mature leaf, root and 7-day-old seedlings was carried out by using GeneChip^® ^Rice Genome Arrays. The raw data from 51 chips representing three biological replicates each from 17 samples was normalized by **G**ene **C**hip **R**obust **M**ultiarray **A**nalysis (GCRMA) algorithm [32]. Since five tandemly duplicated Mγ genes *OsMADS81, 82, 83, 85 *and *99 *showed very high sequence identity (94.6 to 98.6%), the unique probe sets for these genes were not available on the GeneChip^®^. Therefore, a cumulative expression profile for these genes is presented (Figure 4). Expression profiles for *OsMADS78 *and *79*, which were also not represented on the chip, were studied using QPCR (quantitative real-time PCR, Figure 4). The primers used in this study are listed in supplementary table S2 [see Additional file 5]. The initial analysis revealed all but one (*OsMADS80*) gene to be expressing in at least one of the experimental stages analyzed (Figure 4).

**Table 2:** Panicle and seed developmental stages as well as stress treatments used in this study (DAP, Days After Pollination).

**S. No.**	**Symbols Used**	**Developmental stages analyzed**
1	ML	Mature Leaf (collected before pollination)
2	R	Roots of 7-day old seedling
3	SD	7-day old seedling
4	P1	0–3 cm panicle
5	P2	3–5 cm panicle
6	P3	5–10 cm panicle
7	P4	10–15 cm panicle
8	P5	15–22 cm panicle
9	P6	22–30 cm panicle
10	S1	0–2 DAP
11	S2	3–4 DAP
12	S3	5–10 DAP
13	S4	11–20 DAP
14	S5	21–29 DAP
15	CS	Cold Stress
16	DS	Dehydration Stress
17	SS	Salt Stress

*Molecular wt and PI of OsMADS3 has been calculated using Gene Runner

** TIGR Model Represents only K box protein.

**Figure 5:**
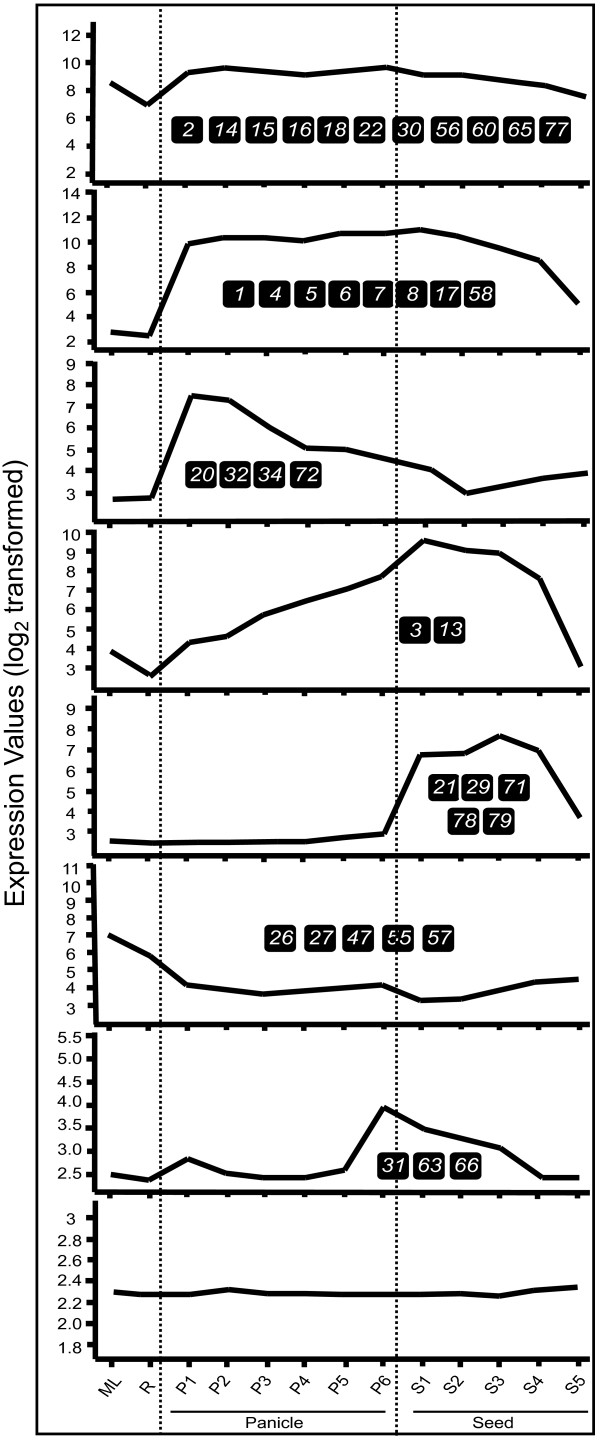
**Expression patterns of MADS-box genes in rice in vegetative as well as panicle and seed development**. Expression profiles have been generated using avadis™ software version 4.2. X-axis represents the developmental stages. Y-axis represents average log_2 _expression values. Genes exhibiting these expression patterns have been represented by numbers. Dotted lines have been drawn to demarcate vegetative organs, panicle and seed developmental stages.

Based on expression profiles during panicle, seed and vegetative development, MADS-box genes were classified into eight groups. Figure 5 shows mean expression profiles for each of the groups. Group I consisted of 12 genes (*OsMADS2, 14, 15, 16, 18, 22, 30, 56, 60, 64, 65 *and *77*) most of which showed high transcript accumulation in all the stages analyzed. However, eight genes (*OsMADS1, 4. 5, 6, 7, 8, 17 *and *58*) of group II showed high expression preferentially in panicle and seed with more than 100 to 500 folds expression in majority of the reproductive tissues in comparison to mature leaf. *OsMADS7 *and *8 *were the most highly expressed genes in panicle and seed stages with more than 1000-fold transcript accumulation in S1 stage of seed development. Group III comprised of the genes *OsMADS20, 32, 34 *and *72*, which showed high expression during early stages of panicle development. The expression declined gradually as the panicles matured. In contrast, the expression of group IV genes (*OsMADS3 *and *13*) increased with the development of the panicles and continued to increase during stages of seed development; the highest expression being 10 and 100 folds for *OsMADS3 *and *13*, respectively. Five genes, *OsMADS21, 29*, *71*, *78 *and *79 *(group V), expressed preferentially during seed development. The expression of *OsMADS21 *and 29 was more than 100 folds in S1-S3 stages, whereas, the expression of *OsMADS71 *increased up to 15 folds in S3 to S5 stages. *OsMADS78 *and *79 *transcripts showed S3-S4 stage-specific accumulation (Figure 4). Group VI comprised of five genes, *OsMADS26, 27, 47, 55 *and *57 *that expressed predominantly in vegetative tissues. The maximum expression of *OsMADS57 *was observed in mature leaves, whereas, *OsMADS27 *showed higher expression in roots as well. Three genes (*OsMADS31, 63 *and *66*) that constituted Group VII showed low levels of expression in most stages of panicle and seed development. The peak expression for these genes was observed either in P6 (*OsMADS63*), S1/S2 (*OsMADS31*) or S3 (*OsMADS66*) stages. The remaining genes (mainly M-type) showed very low levels of expression and were clustered together as group VIII (Figure 4). For normalized expression values and calculated fold change values with respect to mature leaf refer to supplementary tables S3 and S4 [see Additional file 6 and 7].

**Figure 6:**
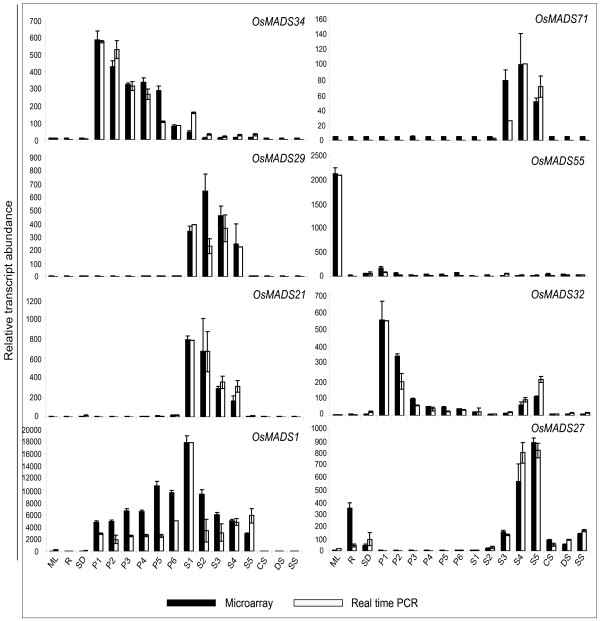
**QPCR results for selected eight genes and its correlation with microarray data**. Two and three biological replicates have been taken for QPCR and microarray, respectively. Standard error bars have been shown for data obtained using both the techniques. Y-axis represents raw expression values obtained using microarays, QPCR data has been normalized to ease profile matching with that of microarrays. X-axis depicts developmental stages as explained in table 2.

**Figure 7:**
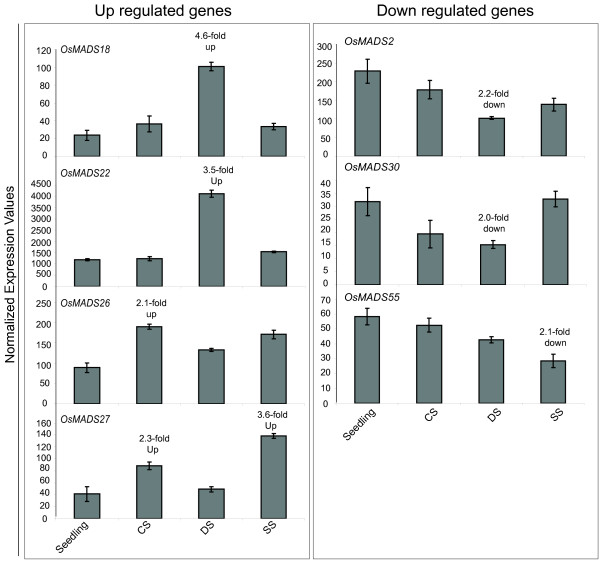
**Differential expressions shown by seven MADS-box genes in response to various abiotic stress conditions**. Left panel shows four genes up regulated and right panel shows down regulated genes more than 2 folds with p value less than 0.05 in response to three abiotic stress conditions. X-axis represents seedling followed by stress samples (CS, cold stress; DS, dehydration stress; SS, salt stress). Y-axis represents average expression values obtained using microarrays. Error bars represent standard error for data obtained in three biological replicates.

Eight genes showing discrete expression patterns were selected for validation by QPCR analysis. Figure 6 shows a comparison of the QPCR and microarray analysis. The expression patterns obtained for all eight genes using QPCR were similar to that derived from the microarrays. Moreover, most of the characterized genes showed similar expression patterns to that already described in the literature, which strengthens the reliability of the data obtained by using microarrays [33]Expression levels of four MADS-box genes (*OsMADS18*, *22*, *26 *and *27*) were up regulated by more than two folds in response to cold and dehydration stress treatments (Figure 7). Three genes (*OsMADS2*, *30 *and *55*) showed more than 2-fold down regulation in response to dehydration and salt stress. The fold change values with respect to seedlings are given in supplementary table S5 [see Additional file 8]

### Expression profiles of putative orthologs of Arabidopsis MIKC^c^-type genes in rice

**Figure 8:**
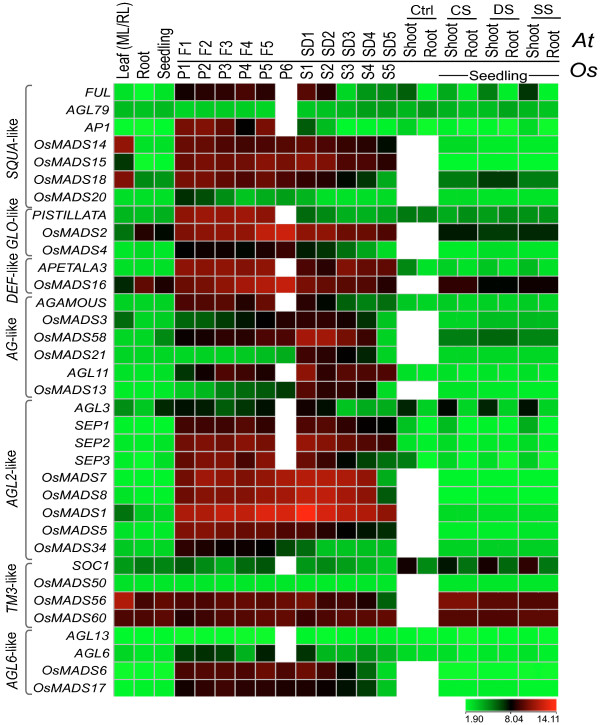
**Expression profiles of selected rice and Arabidopsis MADS-box genes**. (Color bar at the base represents log_2 _expression values, thereby green color representing low level expression, black shows medium level expression and red signifies high level expression). Expression profiles of selected MADS-box genes showing high sequence similarity and similar expression pattern in comparable stages. Developmental stages from *Oryza sativa *(*Os*) and *Arabidopsis thaliana *(*At*) used for expression profiling are mentioned on top of each column listed in the temporal order of development. On the left side of expression map, clade names are given. Developmental stages used for *Arabidopsis *are as follows: Leaf, 21 day rosette leaf (RL); Root, roots of 7-day old seedling; Seedling, 7-day old seedlings; F1-F5, flower stage 9, 10/11, 12, 15 and 28 day mature flower; SD1-SD5, seed stage 3, 5, 7, 9 and 10. For Stress, shoots and roots of 16-day-old seedling subjected to cold/drought/salt stress for 4 hours were selected.

Comparative expression profiles of phylogenetically related MIKC^c ^class genes in rice and *Arabidopsis *is shown in figure 8. These genes have been considered orthologs in *Arabidopsis *and rice [34-36]. In GLO-like clade, the expression of *PI *and *OsMADS4 *was found to be restricted to reproductive tissues, whereas, *OsMADS2 *showed significant expression in seedlings as well. The expression of another B-class gene, *AP3*, was also found to be specific to reproductive tissues in *Arabidopsis*; however its rice ortholog, *OsMADS16*, also expressed in vegetative tissues besides showing peak expression in reproductive tissues. The expression of *AG *was found to be restricted to the stages of floral development and initial stages of seed development in *Arabidopsis*. In rice, its putative orthologs *OsMADS3 *and *OsMADS58 *were found to have comparable expression profiles to that of *AG*, with *OsMADS3 *showing relatively low level transcript accumulation. In *AG *clade, the expression profiles of an *Arabidopsis *D-class gene, *AGL11 *and its rice counterpart *OsMADS13 *showing 53% identity at amino acid level were also comparable. *SUPPRESSOR OF CONSTANS1 *(*SOC1*) and its putative ortholog *OsMADS50 *in ricewere found to exhibit low level ubiquitous expression in the stages analyzed. The members of *AGL2*-like clade, *OsMADS7/45 *and *OsMADS8/24 *are duplicated genes with high level of sequence homology to *AGL2/SEP2 *and *AGL14/SEP1*, respectively. These genes, along with *OsMADS1, 5 *and *34 *were found to exhibit similar expression patterns as those of *SEP *genes. In *SQUA*-like clade, *AP1 *gene of *Arabidopsis *and its putative rice orthologs *OsMADS14 (RAP1B)*, *15(RAP1A) *and *OsMADS18 *show high sequence similarity. In reproductive tissues, the expression profiles of *OsMADS14, 15 *and *18 *were found to be very similar to that of *AP1*, but unlike *AP1*, the rice genes also expressed in mature leaves.

In *AGL6*-like clade, duplicated genes, *OsMADS6 *and *OsMADS17*, exhibit similar expression patterns as that of *AGL6*. Both the genes show more than 50% identity with *AGL6 *at amino acid level suggesting that these could be putative orthologs of *AGL6 *in rice.

### Expression profiles of duplicated genes

**Figure 9:**
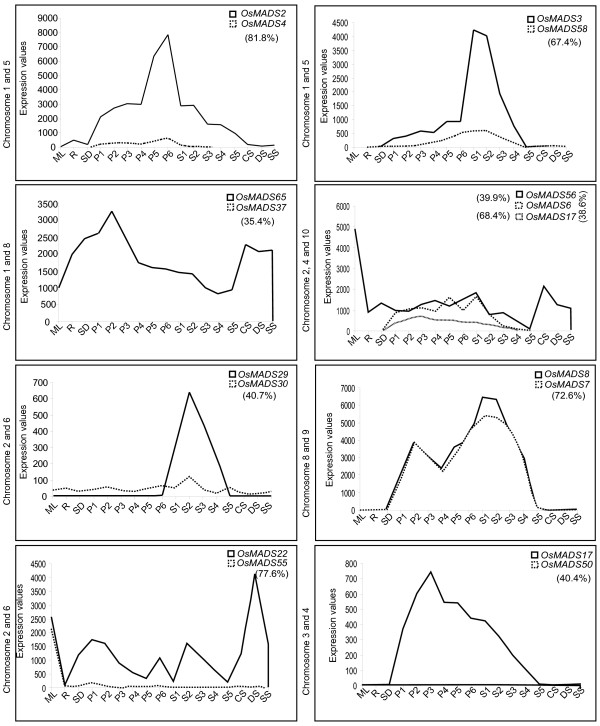
**MADS-box genes lying on duplicated segments of rice genome and their expression pattern**. Expression patterns of duplicated genes have been compared in this figure. X-axis represents the developmental stages as given in table 2. Y-axis represents the raw expression values obtained using microarrays. Chromosome numbers on the left of each graph represent the duplicated segments on which the duplicated genes were identified. Homology for each duplicated gene pair is given in brackets.

Analysis of the TIGR rice segmental duplication database revealed 19 MADS-box genes that were localized on the duplicated segments of the rice genome. A comparison of expression profiles of the duplicated gene pairs, as obtained from microarray data, revealed that except for 3 gene pairs, viz. *OsMADS7:8, OsMADS3:58 *and *OsMADS2:4*; expression patterns of other duplicated genes had diverged significantly (Figure 9).

Chromosome 1 was found to have four tandemly duplicated genes within a 12 kb region. Two groups of tandemly duplicated genes with three genes each were localized on chromosome 4. Incidentally, this region overlapped with an intra-chromosomal duplicated segment suggesting that these six genes probably evolved from a single ancestral gene by a combination of segmental and tandem duplication events. All four tandemly duplicated MIKC^c^-type genes showed varied expression patterns. *OsMADS13 *and *33 *as well as *OsMADS14 *and *34*, although fulfilling our selection criteria, were not considered as being tandemly duplicated because of the high level of sequence divergence, which was evident from their placement in different clades of MIKC^c^-type genes.

## Discussion

### Involvement of MADS-box genes in panicle and seed development in rice

For over a decade, investigations leading to the understanding of genetic and molecular basis of floral development in model eudicots, *Arabidopsis *and *Antirrhinum*, have revealed involvement of a number of MADS-box genes in specifying floral organ identity [37,38]. Attempts have been made to predict the function of MADS-box genes in diverse species based on sequence similarities [24,39]. However, identification of additional paralogs with very similar sequences and existence of duplicated genes with different expression patterns made it difficult to predict the function based on sequence data alone. Similarity in temporal and spatial expression patterns in combination with the sequence comparisons, however, was found to be a better criterion for establishing orthologous relationships. In this paper, we have presented a comprehensive expression profiling for all the MADS-box genes in rice along with an account of their phylogenetic relationships with the *Arabidopsis *genes.

Of the 75 genes analyzed in this study, more than 20 were found to exhibit either specific or preferential transcript accumulation during stages of panicle and seed development. Some of these genes have already been characterized as orthologs of *Arabidopsis *ABCDE class genes, viz.*OsMADS14 and 15 *are *APETALA1 *orthologs; *OsMADS2 *and *4 *are *PISTILLATA *orthologs; *OsMADS16 *is an *APETALA3 *ortholog; *OsMADS3 *and *58 *are *AGAMOUS *orthologs; D class gene, *OsMADS13 *is putative ortholog of *AGL11*; *OsMADS7 *and *8 *are orthologous to *SEPALLATA2 *and *1*, respectively [40-48]. Most of the putative orthologous genes in rice and *Arabidopsis *exhibit similar expression patterns (Figure 8). It was, however, observed that the rice counterparts had a general tendency to express in vegetative organs as well, whereas, the expression of *Arabidopsis *genes was restricted to reproductive tissues. From the expression data and per cent identity, it seems that duplicated genes, *OsMADS6 *and *OsMADS17 *(*AGL6*-like clade) are orthologous to *AGL6*. Further experimentation would be required to verify if these genes have similar functions as well.

In addition to some of the well characterized genes described above, there are several others, e.g. *OsMADS34, 32, 20, 72, 63, 98, 89, 92 *and *86*, that show specific up-regulation in panicles but have not yet been functionally validated in rice. This list also includes MIKC*- and M-type genes along with MIKC^c ^genes. Most of the functionally validated MADS-box family genes belong to the MIKC^c ^class, while functions of most of the M-type genes are not yet known in any system. Therefore, this study provides a solid base to select genes for functional validation.

In 2003, Parenicova and coworkers showed that 64 of the 109 *Arabidopsis *MADS-box genes expressed in siliques [6]. Later, by using high-density transcription factor filter arrays, almost all the MADS-box genes were found to express during silique development [49]. These results suggested that besides being involved in the development of flowers, the MADS-box gene family could be involved in the process of seed development as well. In rice, we have also found (with the exception of *OsMADS80*)that transcripts for almost all the MADS-box genes are expressed in at least one of the seed development stages analyzed. Interestingly, the highest expression values for *OsMADS13, 21, 23, 29, 71, 75, 78 *and *79 *were observed for seed stages, suggesting that these genes could be involved in development of seeds. Four of these genes belong to type II (MIKC^c ^group), whereas, the remainder are type I (Mα-type). Since, two of the type I (Mγ) genes, *AGL80 *and *PHERES*, have previously been implicated in seed development, it might be interesting to investigate the role of other type-I genes showing up-regulation during development of seeds [50,51].

### Rice MIKC^c ^genes

The MIKC^c ^genes have been sub-grouped into 13 clades in *Arabidopsis*. Representatives of all but the *FLC *clade were also found in rice. Six genes belonging to the *FLC *clade have been implicated in control of flowering by vernalization and autonomous pathways in *Arabidopsis*. Since rice does not require vernalization for flowering, this clade has been suggested to be lost in rice [19]. Recently, Zhao and coworkers (2006) have reported a new monocot-specific clade, *OsMADS32*-like clade, consisting of *OsMADS32 *of rice and *TaAGL14 *and *15 *of wheat [20]. The expression of *TaAGL14 *and *15 *was detected in most vegetative stages along with inflorescence and seeds. In contrast, the *OsMADS32 *transcripts were found to be restricted to early stages of panicle and late seed development, suggesting that the *OsMADS32*-like clade might have evolved to cater for diverse monocot-specific functions. A comparison of phylogenetic relationships and expression profiles between rice and *Arabidopsis *MADS-box genes suggests that although most of the basic ABCDE functions have been retained in rice, acquisition of new functions and subfunctionalization of existing gene functions is also apparent.

### Mβ-like genes are represented in rice

In earlier studies, no gene of rice could be assigned to the Mβ group of M-type MADS-box genes, hence it was suggested that probably Mβ genes have not been retained in the rice genome [6,28]. In this study, we have identified nine new genes that grouped with *Arabidopsis *Mβ type genes. Although bootstrap values are low, separation of this clade from the rest of M-type genes of rice and the presence of conserved motifs in *Arabidopsis *Mβ protein and the newly identified group is suggestive of the existence of Mβ group in rice as well.

### Duplication seems to have played major role in diversification of MADS-box family of genes

*Arabidopsis *has been reported to have 107 MADS-box genes [6]. However, in rice that has a genome size almost three times as that of *Arabidopsis *[52,53], the number of MADS-box genes was found to be only 75. The reason for this could be the variable status of whole genome duplications in *Arabidopsis *and rice [54,55]. Surprisingly, however, the number of MIKC^c^-type genes in both *Arabidopsis *and rice was found to be similar at 39 and 38, respectively. Therefore, the difference in the total number is mainly due to the variation in the number of M-type genes, which are 37 in rice and 68 in *Arabidopsis*. It seems that duplication events have contributed significantly towards evolution of M-type genes. Our analysis revealed 16 M-type genes, which could have originated because of tandem duplications (Figure 1). Phylogenetic analysis suggests that rice and *Arabidopsis *Mγ genes probably had a common ancestor and the expansion occurred independently after divergence of monocots and dicots.

MADS-box genes seem to have evolved mainly through gene duplication events followed by neofunctionalization, subfunctionalization or in some cases pseudogenization of the duplicated gene [56]. However, redundancy being one of the fates of duplication is also common in MADS-box family. We found 30 MADS-box genes lying on segmental duplicated regions of rice chromosomes while only 16 were found to have been retained, suggesting that considerable changes may have taken place following segmental duplication leading to loss of some of the genes. Except one, all paralogous gene pairs belong to MIKC^c^-type of MADS-box family. Expression data show that most of these duplicated genes have divergent expression patterns that may be because they have undergone neofunctionalization or subfunctionalization, though sufficient experimentation is required to prove this hypothesis. Interestingly, three genes, viz. *OsMADS6, 17 *and *56*, lying on duplicated segments of chromosomes 2, 4 and 10, respectively, show collinearity in gene order. On the other hand, *OsMADS50 *lying on chromosome 3 shows synteny with only one of these genes, i.e. *OsMADS17*. They may all have resulted due to duplication of a segment on chromosome 4, but thereafter, evolution of these genes may have been quite independent resulting in loss of micro-collinearity between the duplicated regions.

### Stress responsive MADS-box genes in rice

MADS-box genes have been shown to be affected by low temperature stress in tomato [57] and by application of hormones like cytokinins, gibberellins [58], ethylene [59] and auxins [60] in other plants. Seven MADS-box genes exhibited differential expression in response to cold, salt and/or desiccation stress in rice. So far, none of these genes has been implicated in stress response. Amongst stress-induced genes, *OsMADS18 *is a member of *AP1/SQUA *group that has been shown to express widely during development with its transcripts accumulating at high levels specifically in meristematic tissues [61]. It has been shown to interact with *OsMADS6*, *8/24*, *7/45*, and *47 *[45,61] and in our analysis its expression pattern was found to overlap with those of *OsMADS6, 8 *and *7 *in reproductive tissues and with *OsMADS47 *during vegetative development, suggesting that it might be interacting with different partners during reproductive development and stress. Recently, Tardif and coworkers showed that a large number of genes involved in flower development are associated with abiotic stress responses in wheat [62]. Our preliminary analysis involving transcript profiling during reproductive development and abiotic stress conditions has also revealed approximately 400 genes that are up regulated during panicle/seed development and three stress conditions, viz. cold, salt, and dehydration (unpublished data). It would be, therefore, interesting to undertake specific investigations, which could establish the interactions of biochemical pathways that are activated during reproductive development and stress response.

## Conclusion

Contribution of MADS-box gene family in flower organ specification is well documented in eudicots; however, functions of many gene members of this class have not been elucidated in rice. A comparison of phylogenetic relationships and expression profiles between rice and *Arabidopsis *MADS-box genes suggests that although most of the basic ABCDE functions have been retained in rice, acquisition of new functions and subfunctionalization of existing gene functions is also not uncommon. Furthermore, the role of MADS-box transcription factors in seed development and during stress response also needs to be explored. The new information generated is expected to help in selection of appropriate candidate genes for further functional characterization.

## Methods

### Identification of genes, nomenclature and mapping on chromosomes

Name Search and Hidden Markov Model (HMM) were employed to identify the MADS-box genes from rice genome. MADS-box sequences available for all land plants were downloaded from SWISSPROT and TrEMBL [63] and their HMM profile was generated using HMMER 2.1.1 software package [64,65]. This profile was used to search the complete proteome of rice available in TIGR [66] and KOME [67,68] databases using Basic Local Alignment Search Tool (BLAST; [69]) with filter off. Name search using MADS, SRF, AGAMOUS and AP1 as keywords in these databases helped in identification of more genes, which could not be identified using HMM profile due to the presence of incomplete MADS-box. Redundant sequences were removed by aligning the protein sequences using ClustalX 1.83 [70] and checking their genomic locus in TIGR. Motif scan was performed using SMART [71,72] or National Center for Biotechnology Information Conserved Domain Database (NCBI-CD; [73]) searches with filter off. According to already available nomenclature, 34 MADS-box genes have been named from *OsMADS1 *to *58*. Thus, the newly identified genes were named from *OsMADS59 *to *99*. MADS-box genes were mapped on chromosomes by identifying their chromosomal position given in the TIGR rice database. Information regarding ORF length, amino acids number, molecular weight and isoelectric point of protein was downloaded from TIGR release 4. For *OsMADS3 *and *20*, Gene Runner program version 3.04 was employed to find molecular weight and PI of protein, as it was not available in TIGR.

### Phylogenetic analysis

To identify the number of groups formed by rice MADS-box genes in comparison to *Arabidopsis*, MADS-box domain comprising of 60 amino acids, identified by SMART from all the MADS-box sequences of *Arabidopsis *and rice were aligned using ClustalX (version 1.83) program. An un-rooted neighbor-joining (NJ; [74]) phylogenetic tree was constructed in ClustalX with default parameters. Separate phylogenetic trees were constructed using complete protein sequence and coding sequences of rice and *Arabidopsis *MADS-box genes. Bootstrap analysis was performed using 1000 replicates. The trees thus obtained were viewed using TREEVIEW software [75].

### Sequence and duplication analysis

To identify the conserved motifs, MEME version 2.2 [76] was employed using following parameters; number of repetitions – any, maximum number of motifs – 20, optimum motif width set to ≥ 6 and ≤ 200. The motifs obtained were annotated using SMART and NCBI-CD search program.

Further, MADS-box gene duplications were mapped on segmental duplications database of TIGR with 100 kb as well as 500 kb distance allowed between collinear genes [66]. For finding tandemly duplicated candidates, genes with intergenic distance not more than 20 kb and having fair degree of overall homology between them were selected. Identity among duplicated genes was calculated using DNASTAR MegAlign 4.03 package.

### Collection of plant material

*Oryza sativa indica *var. IR64 tillers spanning all stages of panicle and seed development were collected from field grown rice. Mature leaves were also harvested from same plants. For all the stages, three biological replicates were harvested from independent populations of plants. After harvesting, panicle and seed samples were frozen in liquid nitrogen and stored at -70°C. For stress treatment, rice seeds were surface-sterilized with 0.1% HgCl_2 _and soaked in RO (reverse osmosis) water overnight in dark. Next day, the seeds were spread on a meshed float and grown hydroponically at 28 ± 1°C in culture room conditions. After 7 days of growth, the seedlings were transferred to 100 ml beaker for treatment. For salt stress, sodium chloride was used at final concentration of 200 mM for 3 hours. For cold stress, seedlings were kept at 4°C for 3 hours. Desiccation stress was simulated by drying the plants on tissue paper and spreading them on Whatmann 3 mm sheet for 3 hours. The seedlings with their roots kept in water, for 3 hours duration, were used as control.

### Microarray experiments

Affymetrix GeneChip^® ^Rice Genome Arrays representing 49,824 transcripts (48,564 of *japonica *and 1,260 of *indica*) have been employed to study the transcriptome profiles of MADS-box genes during reproductive organ development and stress response in rice. Total RNA was isolated from all the tissues, except seeds, using TRIzol method (Invitrogen Inc., USA; [77]). Due to high carbohydrate content, RNA from seed samples was isolated using the method described earlier [78]. After checking the quality on agarose formaldehyde or TAE gels, the RNA samples were quantified using nanodrop (ND-1000 Spectrophotometer). Five micrograms of RNA with 260:280 ratios of 1.9–2.0 and 260:230 ratios more than 2.0 was used for cDNA synthesis. Labeling and hybridizations were carried out according to Affymetrix manual for one-cycle target labeling (Affymetrix, Santa Clara, CA). Hybridization was performed in GeneChip^® ^Hybridization Oven 640 for 16 hours at 45°C and 60 rpm. GeneChips were washed and stained with streptavidin-phycoerythrin using the fluidics protocol EukGE_WS2V5_450 in Affymetrix fluidic station model 450. Finally, chips were scanned using the GeneChip^® ^Scanner 3000.

### Digital expression analysis

The expression data for *Arabidopsis*, using Affymetrix GeneChip^® ^ATH1 Genome Array, from stages comparable to those used for rice was obtained from Gene Expression Omnibus (GEO) database at the NCBI under the series accession numbers GSE5620, GSE5621, GSE5623, GSE5624, GSE5629, GSE5630, GSE5631, GSE5632 and GSE5634. Total of 55 CEL files representing 21 stages of development as well as stress treatments were downloaded from [79] and analyzed by using avadis™ microarray data analysis software version 4.2 [80]. The data was normalized using GCRMA followed by log transformation and average calculation. Heat Map was generated for selected genes.

### Microarray data analysis

CEL files generated in GeneChip Operating Software (GCOS) were further analyzed using avadis™. Data were normalized using GCRMA algorithm and log transformed. To get the expression values, averages of three biological replicates were used. The expression data for MADS-box genes was extracted by using name search and the gene IDs listed in table 1. Wherever more than one probe set was available for one gene, the probe set designed from 3' end was given preference. Cluster analysis on rows was performed using Euclidean distance metric, and Ward's Linkage rule of Hierarchical clustering. Differential expression analysis was performed taking mature leaf as reference to identify genes expressing more than two folds in panicle and seed, with p values <0.005. Similarly, for identifying stress-induced genes, differential expression analysis was performed with no correction applied and p values less than 0.05. Further K-means clustering was performed to identify the expression patterns shown by genes expressing in panicle and seed. Since 73 genes (69 probe sets) are represented on chip, expression profiles of *OsMADS78 *and *79 *were studied using QPCR. Raw microarray data have been deposited in the Gene Expression Omnibus database at the National Center for Biotechnology Information under the series accession numbers GSE6893 and GSE6901.

### QPCR

Real time PCR reactions were carried out using the same RNA samples, which were used for microarrays as described earlier [81]. In brief, primers were designed for all the genes preferentially from 3' end of the gene using PRIMER EXPRESS version 2.0 (PE Applied Biosystems, USA) with default parameters. Each primer was checked using BLAST tool of NCBI database with filter off for its specificity for respective gene, which was further confirmed by dissociation curve analysis obtained after the PCR reaction. First strand cDNA was synthesized by reverse transcription using 4 μg of total RNA in 100 μl of reaction volume using high-capacity cDNA Archive kit (Applied Biosystems, USA). Diluted cDNA samples were used for Real time PCR analysis with 200 nM of each primer mixed with SYBR Green PCR master as per manufacturer's instructions. The reaction was carried out in 96-well optical reaction plates (Applied Biosystems, USA), using ABI Prism 7000 Sequence Detection System and software (PE Applied Biosystems, USA). To normalize the variance among samples, *Actin *was used as endogenous control. Relative expression values were calculated after normalizing against the maximum expression value. These data were further normalized to ease the profile matching to that obtained from microarrays.

## Authors' contributions

RA has done the computational analysis, microarray experiments for mature leaf and panicle stages, performed real-time PCR, analyzed the data and drafted the manuscript. PA has performed microarray for root and seed stages. SR has performed microarray experiments for seedling and stress stages. AKS and VPS had grown the rice plants, provided tissue material, and helped in identification of biological stages for microarray experiments. AKT was involved in planning of experiments, revised the final version of the manuscript and headed the project. SK designed and participated in all the experiments and revised the final text of the manuscript. All authors have read and approved the final manuscript.

## Supplementary Material

Additional file 1 **Figure S1. Phylogenetic analysis of 173 MADS-box domain sequences from rice (75) and Arabidopsis (98)**. ~60 residues of conserved MADS-box domain are used for constructing the phylogenetic tree. *Arabidopsis *genes are marked by A followed by number. Similarly rice genes are marked by O followed by number assigned to each gene. Due to shorter branch lengths of MIKC^c^-type genes, a magnified view is presented for this group to mark the genes. Click here for file

Additional file 2 **Figure S2. Phylogenetic relationships among rice and Arabidopsis MADS-box proteins**. An unrooted tree has been drawn using complete MADS-box proteins of rice and *Arabidopsis*. Bootstrap values from 1000 replicates have been indicated at nodes. Scale bar represents 0.1 amino acid substitution per site. Click here for file

Additional file 3 **Figure S3. Phylogenetic relationships among rice and Arabidopsis MADS-box genes**. An unrooted NJ (neighbor-joining) tree based on all the nucleotide substitutions in coding exons of rice and *Arabidopsis *MADS-box genes. Bootstrap probabilities from 1000 replicates have been indicated at nodes. The genes showing different topology in DNA-based tree in comparison to protein-based tree have been marked by *. Scale bar represents 0.1 substitutions per nucleotide position. Click here for file

Additional file 4 Table S1. Sequence and length of motifs identified from rice MADS-box proteins using MEME motif search tool (aa, amino acids). Click here for file

Additional file 5 Table S2. List of primers used for QPCR. Click here for file

Additional file 6 Table S3. Normalized expression values obtained for all MADS-box genes in microarrays. Click here for file

Additional file 7 **Table S4. Results of differential expression analysis taking mature leaf as reference**. Fold changes have been taken with p value less than or equal to 0.005. Click here for file

Additional file 8 Table S5. Results obtained after differential expression analysis to extract stress responsive genes using microarrays (seedling taken as reference). Click here for file

## References

[B1] de Folter S, Angenent GC (2006). trans meets cis in MADS science. Trends Plant Sci.

[B2] Jack T (2001). Plant development going MADS. Plant Mol Biol.

[B3] Kaufmann K, Melzer R, Theissen G (2005). MIKC-type MADS-domain proteins: structural modularity, protein interactions and network evolution in land plants. Gene.

[B4] Nam J, dePamphilis CW, Ma H, Nei M (2003). Antiquity and evolution of the MADS-box gene family controlling flower development in plants. Mol Biol Evol.

[B5] Ng M, Yanofsky MF (2001). Function and evolution of the plant MADS-box gene family. Nat Rev Genet.

[B6] Parenicova L, de Folter S, Kieffer M, Horner DS, Favalli C, Busscher J, Cook HE, Ingram RM, Kater MM, Davies B, Angenent GC, Colombo L (2003). Molecular and phylogenetic analyses of the complete MADS-box transcription factor family in Arabidopsis: new openings to the MADS world. Plant Cell.

[B7] Purugganan MD (1997). The MADS-box floral homeotic gene lineages predate the origin of seed plants: phylogenetic and molecular clock estimates. J Mol Evol.

[B8] Theissen G, Becker A, Di Rosa A, Kanno A, Kim JT, Munster T, Winter KU, Saedler H (2000). A short history of MADS-box genes in plants. Plant Mol Biol.

[B9] Passmore S, Maine GT, Elble R, Christ C, Tye BK (1988). Saccharomyces cerevisiae protein involved in plasmid maintenance is necessary for mating of MAT alpha cells. J Mol Biol.

[B10] Yanofsky MF, Ma H, Bowman JL, Drews GN, Feldmann KA, Meyerowitz EM (1990). The protein encoded by the Arabidopsis homeotic gene agamous resembles transcription factors. Nature.

[B11] Sommer H, Beltran JP, Huijser P, Pape H, Lonnig WE, Saedler H, Schwarz-Sommer Z (1990). Deficiens, a homeotic gene involved in the control of flower morphogenesis in Antirrhinum majus: the protein shows homology to transcription factors. EMBO J.

[B12] Norman C, Runswick M, Pollock R, Treisman R (1988). Isolation and properties of cDNA clones encoding SRF, a transcription factor that binds to the c-fos serum response element. Cell.

[B13] Yang Y, Fanning L, Jack T (2003). The K domain mediates heterodimerization of the Arabidopsis floral organ identity proteins, APETALA3 and PISTILLATA. Plant J.

[B14] Cho S, Jang S, Chae S, Chung KM, Moon YH, An G, Jang SK (1999). Analysis of the C-terminal region of Arabidopsis thaliana APETALA1 as a transcription activation domain. Plant Mol Biol.

[B15] Egea-Cortines M, Saedler H, Sommer H (1999). Ternary complex formation between the MADS-box proteins SQUAMOSA, DEFICIENS and GLOBOSA is involved in the control of floral architecture in Antirrhinum majus. EMBO J.

[B16] Alvarez-Buylla ER, Pelaz S, Liljegren SJ, Gold SE, Burgeff C, Ditta GS, Ribas de Pouplana L, Martinez-Castilla L, Yanofsky MF (2000). An ancestral MADS-box gene duplication occurred before the divergence of plants and animals. Proc Natl Acad Sci U S A.

[B17] Henschel K, Kofuji R, Hasebe M, Saedler H, Munster T, Theissen G (2002). Two ancient classes of MIKC-type MADS-box genes are present in the moss Physcomitrella patens. Mol Biol Evol.

[B18] Kofuji R, Sumikawa N, Yamasaki M, Kondo K, Ueda K, Ito M, Hasebe M (2003). Evolution and divergence of the MADS-box gene family based on genome-wide expression analyses. Mol Biol Evol.

[B19] Becker A, Theissen G (2003). The major clades of MADS-box genes and their role in the development and evolution of flowering plants. Mol Phylogenet Evol.

[B20] Zhao T, Ni Z, Dai Y, Yao Y, Nie X, Sun Q (2006). Characterization and expression of 42 MADS-box genes in wheat (Triticum aestivum L.). Mol Genet Genomics.

[B21] De Bodt S, Raes J, Florquin K, Rombauts S, Rouze P, Theissen G, Van de Peer Y (2003). Genomewide structural annotation and evolutionary analysis of the type I MADS-box genes in plants. J Mol Evol.

[B22] De Bodt S, Raes J, Van de Peer Y, Theissen G (2003). And then there were many: MADS goes genomic. Trends Plant Sci.

[B23] Alvarez-Buylla ER, Liljegren SJ, Pelaz S, Gold SE, Burgeff C, Ditta GS, Vergara-Silva F, Yanofsky MF (2000). MADS-box gene evolution beyond flowers: expression in pollen, endosperm, guard cells, roots and trichomes. Plant J.

[B24] Riechmann JL, Meyerowitz EM (1997). MADS domain proteins in plant development. Biol Chem.

[B25] Rounsley SD, Ditta GS, Yanofsky MF (1995). Diverse roles for MADS box genes in Arabidopsis development. Plant Cell.

[B26] Saedler H, Becker A, Winter KU, Kirchner C, Theissen G (2001). MADS-box genes are involved in floral development and evolution. Acta Biochim Pol.

[B27] Moore S, Vrebalov J, Payton P, Giovannoni J (2002). Use of genomics tools to isolate key ripening genes and analyse fruit maturation in tomato. J Exp Bot.

[B28] Nam J, Kim J, Lee S, An G, Ma H, Nei M (2004). Type I MADS-box genes have experienced faster birth-and-death evolution than type II MADS-box genes in angiosperms. Proc Natl Acad Sci U S A.

[B29] Furutani I, Sukegawa S, Kyozuka J (2006). Genome-wide analysis of spatial and temporal gene expression in rice panicle development. Plant J.

[B30] Itoh J, Nonomura K, Ikeda K, Yamaki S, Inukai Y, Yamagishi H, Kitano H, Nagato Y (2005). Rice plant development: from zygote to spikelet. Plant Cell Physiol.

[B31] Oryzabase. http://www.shigen.nig.ac.jp/rice/oryzabase/top/top.jsp.

[B32] Wu Z, Irizarry RA, Gentleman R, Murillo FM, Spencer F (2003). A Model Based Background Adjustment for Oligonucleotide Expression Arrays. Technical Report, Department of Biostatistics.. Working Papers, Baltimore, MD.

[B33] Pelucchi N, Fornara F, Favalli C, Masiero S, Lago C, Enrico Pe M, Colombo L, Kater MM (2002). Comparative analysis of rice MADS-box genes expressed during flower development. Sex Plant Reprod.

[B34] Kater MM, Dreni L, Colombo L (2006). Functional conservation of MADS-box factors controlling floral organ identity in rice and Arabidopsis. J Exp Bot.

[B35] Kyozuka J, Kobayashi T, Morita M, Shimamoto K (2000). Spatially and temporally regulated expression of rice MADS box genes with similarity to Arabidopsis class A, B and C genes. Plant Cell Physiol.

[B36] Lee S, Kim J, Han JJ, Han MJ, An G (2004). Functional analyses of the flowering time gene OsMADS50, the putative SUPPRESSOR OF OVEREXPRESSION OF CO 1/AGAMOUS-LIKE 20 (SOC1/AGL20) ortholog in rice. Plant J.

[B37] Coen ES, Meyerowitz EM (1991). The war of the whorls: genetic interactions controlling flower development. Nature.

[B38] Ma H (1994). The unfolding drama of flower development: recent results from genetic and molecular analyses. Genes Dev.

[B39] Kramer EM, Dorit RL, Irish VF (1998). Molecular evolution of genes controlling petal and stamen development: duplication and divergence within the APETALA3 and PISTILLATA MADS-box gene lineages. Genetics.

[B40] Yamaguchi T, Lee DY, Miyao A, Hirochika H, An G, Hirano HY (2006). Functional diversification of the two C-class MADS box genes OSMADS3 and OSMADS58 in Oryza sativa. Plant Cell.

[B41] Greco R, Stagi L, Colombo L, Angenent GC, Sari-Gorla M, Pe ME (1997). MADS box genes expressed in developing inflorescences of rice and sorghum. Mol Gen Genet.

[B42] Jia H, Chen R, Cong B, Cao K, Sun C, Luo D (2000). Characterization and transcriptional profiles of two rice MADS-box genes. Plant Science.

[B43] Kang HG, Jeon JS, Lee S, An G (1998). Identification of class B and class C floral organ identity genes from rice plants. Plant Mol Biol.

[B44] Kyozuka J, Shimamoto K (2002). Ectopic expression of OsMADS3, a rice ortholog of AGAMOUS, caused a homeotic transformation of lodicules to stamens in transgenic rice plants. Plant Cell Physiol.

[B45] Moon YH, Jung JY, Kang HG, An G (1999). Identification of a rice APETALA3 homologue by yeast two-hybrid screening. Plant Mol Biol.

[B46] Prasad K, Vijayraghavan U (2003). Double-stranded RNA interference of a rice PI/GLO paralog, OsMADS2, uncovers its second-whorl-specific function in floral organ patterning. Genetics.

[B47] Lopez-Dee ZP, Wittich P, Enrico Pe M, Rigola D, Del Buono I, Gorla MS, Kater MM, Colombo L (1999). OsMADS13, a novel rice MADS-box gene expressed during ovule development. Dev Genet.

[B48] Chung YY, Kim SR, Kang HG, Noh YS, Park MC, Finkel D, An G (1995). Characterization of two MADS box genes homologous to GLOBOSA. Plant Science.

[B49] de Folter S, Busscher J, Colombo L, Losa A, Angenent GC (2004). Transcript profiling of transcription factor genes during silique development in Arabidopsis. Plant Mol Biol.

[B50] Kohler C, Hennig L, Spillane C, Pien S, Gruissem W, Grossniklaus U (2003). The Polycomb-group protein MEDEA regulates seed development by controlling expression of the MADS-box gene PHERES1. Genes Dev.

[B51] Portereiko MF, Lloyd A, Steffen JG, Punwani JA, Otsuga D, Drews GN (2006). AGL80 is required for central cell and endosperm development in Arabidopsis. Plant Cell.

[B52] International Rice Genome Sequencing Project (2005). The map-based sequence of the rice genome. Nature.

[B53] Vij S, Gupta V, Kumar D, Vydianathan R, Raghuvanshi S, Khurana P, Khurana JP, Tyagi AK (2006). Decoding the rice genome. Bioessays.

[B54] Paterson AH, Bowers JE, Chapman BA (2004). Ancient polyploidization predating divergence of the cereals, and its consequences for comparative genomics. Proc Natl Acad Sci U S A.

[B55] Yu J, Wang J, Lin W, Li S, Li H, Zhou J, Ni P, Dong W, Hu S, Zeng C, Zhang J, Zhang Y, Li R, Xu Z, Li S, Li X, Zheng H, Cong L, Lin L, Yin J, Geng J, Li G, Shi J, Liu J, Lv H, Li J, Wang J, Deng Y, Ran L, Shi X, Wang X, Wu Q, Li C, Ren X, Wang J, Wang X, Li D, Liu D, Zhang X, Ji Z, Zhao W, Sun Y, Zhang Z, Bao J, Han Y, Dong L, Ji J, Chen P, Wu S, Liu J, Xiao Y, Bu D, Tan J, Yang L, Ye C, Zhang J, Xu J, Zhou Y, Yu Y, Zhang B, Zhuang S, Wei H, Liu B, Lei M, Yu H, Li Y, Xu H, Wei S, He X, Fang L, Zhang Z, Zhang Y, Huang X, Su Z, Tong W, Li J, Tong Z, Li S, Ye J, Wang L, Fang L, Lei T, Chen C, Chen H, Xu Z, Li H, Huang H, Zhang F, Xu H, Li N, Zhao C, Li S, Dong L, Huang Y, Li L, Xi Y, Qi Q, Li W, Zhang B, Hu W, Zhang Y, Tian X, Jiao Y, Liang X, Jin J, Gao L, Zheng W, Hao B, Liu S, Wang W, Yuan L, Cao M, McDermott J, Samudrala R, Wang J, Wong GK, Yang H (2005). The Genomes of Oryza sativa: a history of duplications. PLoS Biol.

[B56] Irish VF, Litt A (2005). Flower development and evolution: gene duplication, diversification and redeployment. Curr Opin Genet Dev.

[B57] Lozano R, Angosto T, Gomez P, Payan C, Capel J, Huijser P, Salinas J, Martinez-Zapater JM (1998). Tomato flower abnormalities induced by low temperatures are associated with changes of expression of MADS-Box genes. Plant Physiol.

[B58] Bonhomme F, Kurz B, Melzer S, Bernier G, Jacqmard A (2000). Cytokinin and gibberellin activate SaMADS A, a gene apparently involved in regulation of the floral transition in Sinapis alba. Plant J.

[B59] Ando S, Sato Y, Kamachi S, Sakai S (2001). Isolation of a MADS-box gene (ERAF17) and correlation of its expression with the induction of formation of female flowers by ethylene in cucumber plants (Cucumis sativus L.). Planta.

[B60] Zhu C, Perry SE (2005). Control of expression and autoregulation of AGL15, a member of the MADS-box family. Plant J.

[B61] Fornara F, Parenicova L, Falasca G, Pelucchi N, Masiero S, Ciannamea S, Lopez-Dee Z, Altamura MM, Colombo L, Kater MM (2004). Functional characterization of OsMADS18, a member of the AP1/SQUA subfamily of MADS box genes. Plant Physiol.

[B62] Tardif G, Kane NA, Adam H, Labrie L, Major G, Gulick P, Sarhan F, Laliberte JF (2007). Interaction network of proteins associated with abiotic stress response and development in wheat. Plant Mol Biol.

[B63] UniProt Knowledgebase. http://www.expasy.org/sprot/.

[B64] HMMER. http://hmmer.janelia.org/.

[B65] Madera M, Gough J (2002). A comparison of profile hidden Markov model procedures for remote homology detection. Nucleic Acids Res.

[B66] TIGR Rice Genome Annotation. http://www.tigr.org/tdb/e2k1/osa1/.

[B67] Knowledge-based Oryza Molecular biological Encyclopedia (KOME). http://cdna01.dna.affrc.go.jp/cDNA/.

[B68] Kikuchi S, Satoh K, Nagata T, Kawagashira N, Doi K, Kishimoto N, Yazaki J, Ishikawa M, Yamada H, Ooka H, Hotta I, Kojima K, Namiki T, Ohneda E, Yahagi W, Suzuki K, Li CJ, Ohtsuki K, Shishiki T, Otomo Y, Murakami K, Iida Y, Sugano S, Fujimura T, Suzuki Y, Tsunoda Y, Kurosaki T, Kodama T, Masuda H, Kobayashi M, Xie Q, Lu M, Narikawa R, Sugiyama A, Mizuno K, Yokomizo S, Niikura J, Ikeda R, Ishibiki J, Kawamata M, Yoshimura A, Miura J, Kusumegi T, Oka M, Ryu R, Ueda M, Matsubara K, Kawai J, Carninci P, Adachi J, Aizawa K, Arakawa T, Fukuda S, Hara A, Hashizume W, Hayatsu N, Imotani K, Ishii Y, Itoh M, Kagawa I, Kondo S, Konno H, Miyazaki A, Osato N, Ota Y, Saito R, Sasaki D, Sato K, Shibata K, Shinagawa A, Shiraki T, Yoshino M, Hayashizaki Y, Yasunishi A (2003). Collection, mapping, and annotation of over 28,000 cDNA clones from japonica rice. Science.

[B69] Altschul SF, Madden TL, Schaffer AA, Zhang J, Zhang Z, Miller W, Lipman DJ (1997). Gapped BLAST and PSI-BLAST: a new generation of protein database search programs. Nucleic Acids Res.

[B70] Thompson JD, Gibson TJ, Plewniak F, Jeanmougin F, Higgins DG (1997). The CLUSTAL_X windows interface: flexible strategies for multiple sequence alignment aided by quality analysis tools. Nucleic Acids Res.

[B71] Letunic I, Goodstadt L, Dickens NJ, Doerks T, Schultz J, Mott R, Ciccarelli F, Copley RR, Ponting CP, Bork P (2002). Recent improvements to the SMART domain-based sequence annotation resource. Nucleic Acids Res.

[B72] Schultz J, Milpetz F, Bork P, Ponting CP (1998). SMART, a simple modular architecture research tool: identification of signaling domains. Proc Natl Acad Sci U S A.

[B73] NCBI Conserved Domains Database. http://www.ncbi.nlm.nih.gov/Structure/cdd/wrpsb.cgi.

[B74] Saitou N, Nei M (1987). The neighbor-joining method: a new method for reconstructing phylogenetic trees. Mol Biol Evol.

[B75] Page RD (1996). TreeView: an application to display phylogenetic trees on personal computers. Comput Appl Biosci.

[B76] Bailey TL, Elkan C (1995). The value of prior knowledge in discovering motifs with MEME. Proc Int Conf Intell Syst Mol Biol.

[B77] Chomczynski P, Sacchi N (1987). Single-step method of RNA isolation by acid guanidinium thiocyanate-phenol-chloroform extraction. Anal Biochem.

[B78] Singh G, Kumar S, Singh P (2003). A quick method to isolate RNA from wheat and other carbohydrate-rich seeds.. Plant Mol Biol Rep.

[B79] Gene Expression Omnibus. http://www.ncbi.nlm.nih.gov/geo/.

[B80] Avadis™. http://avadis.strandls.com/.

[B81] Jain M, Kaur N, Garg R, Thakur JK, Tyagi AK, Khurana JP (2006). Structure and expression analysis of early auxin-responsive Aux/IAA gene family in rice (Oryza sativa). Funct Integr Genomics.

