# Supplementary figures and images for: MADS-box gene family in rice: genome-wide identification, organization and expression profiling during reproductive development and stress
Source: BMC Genomics. 2007 Jul 18;8:242. doi: 10.1186/1471-2164-8-242 (PMC1947970; doi:10.1186/1471-2164-8-242)

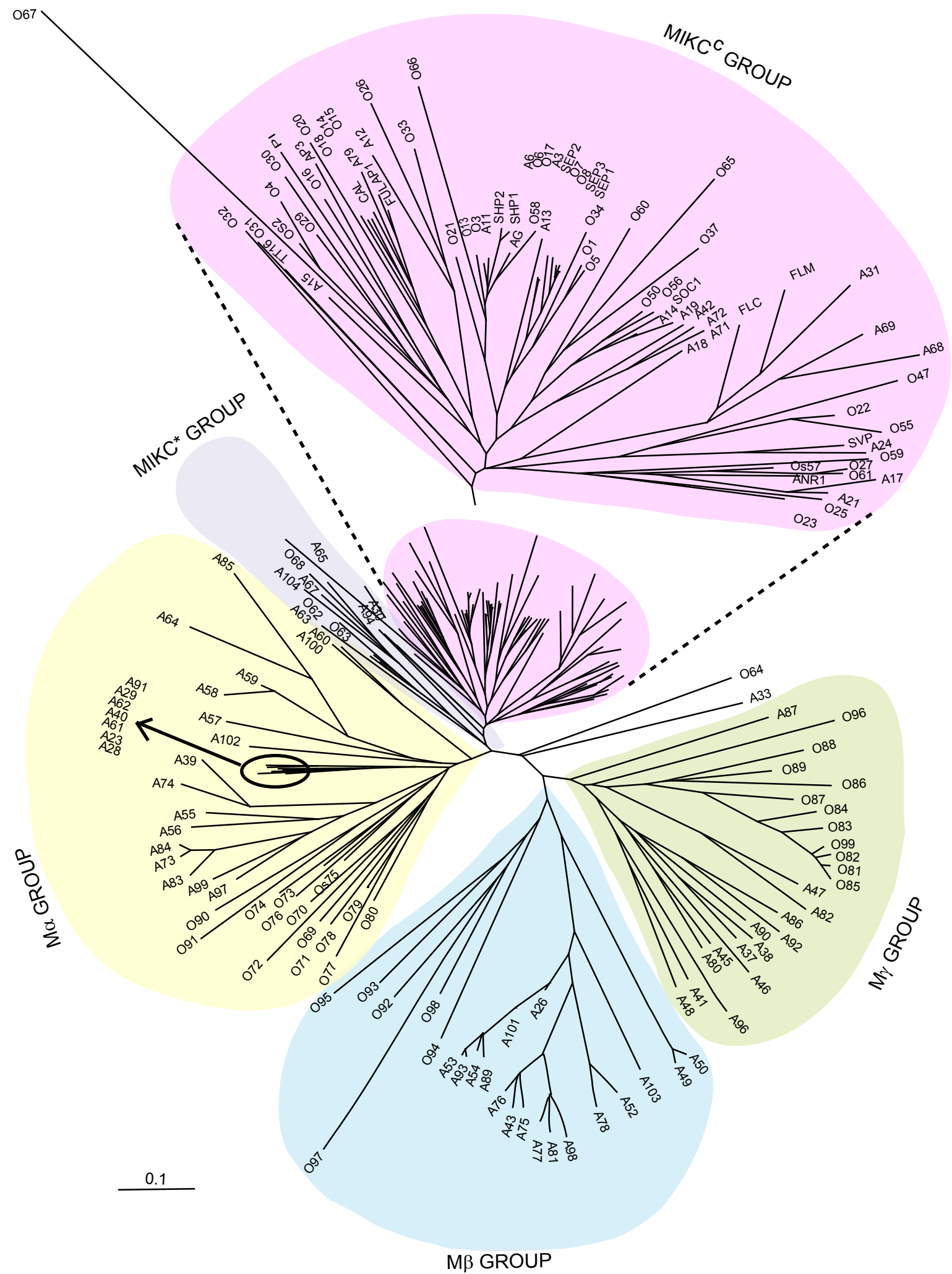

Supplement: Additional file 1 — Figure S1. Phylogenetic analysis of 173 MADS-box domain sequences from rice (75) and Arabidopsis (98). ~60 residues of conserved MADS-box domain are used for constructing the phylogenetic tree. Arabidopsis genes are marked by A followed by number. Similarly rice genes are marked by O followed by number assigned to each gene. Due to shorter branch lengths of MIKCc-type genes, a magnified view is presented for this group to mark the genes. [file 1471-2164-8-242-S1.pdf]

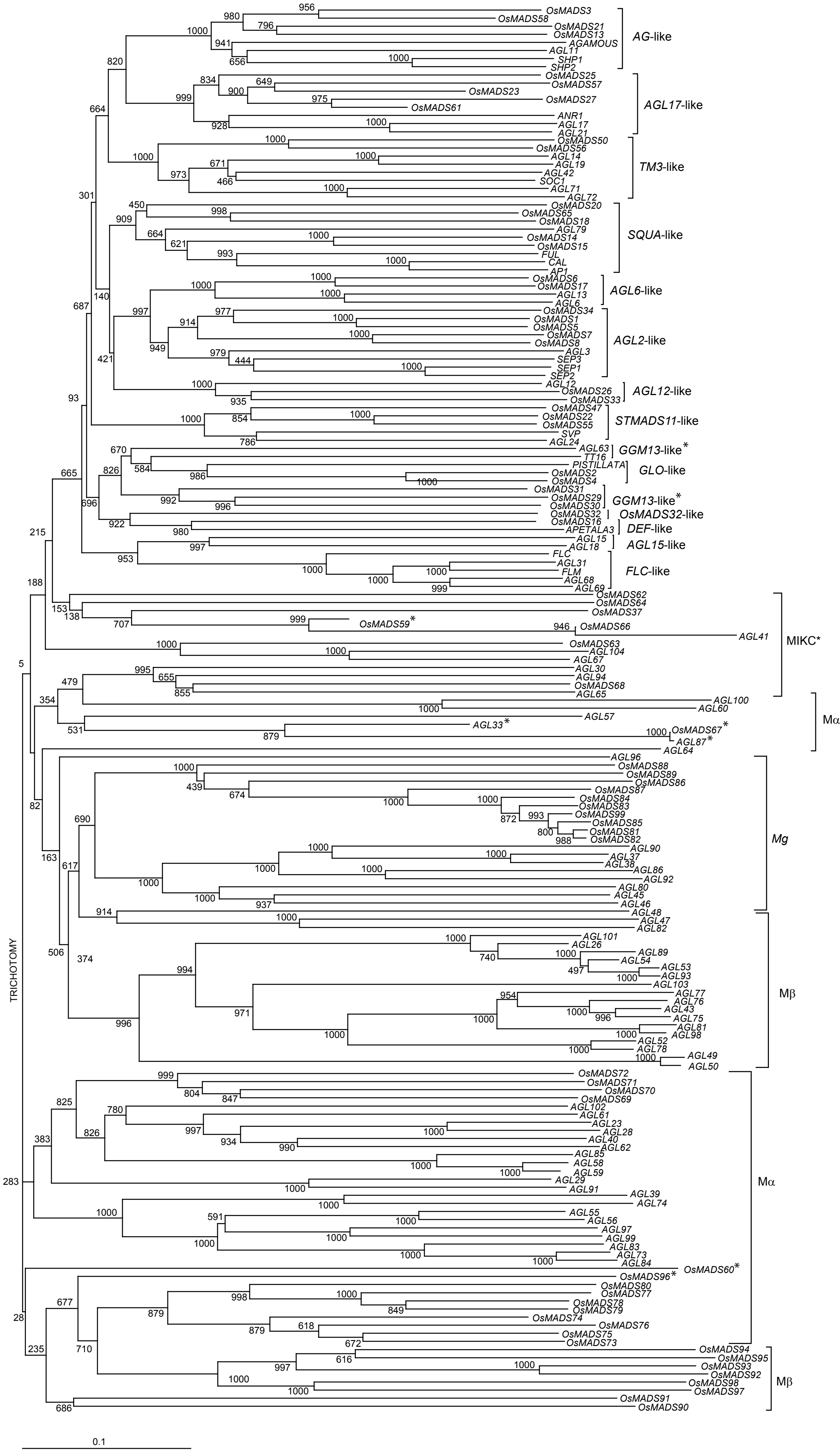

Supplement: Additional file 3 — Figure S3. Phylogenetic relationships among rice and Arabidopsis MADS-box genes. An unrooted NJ (neighbor-joining) tree based on all the nucleotide substitutions in coding exons of rice and Arabidopsis MADS-box genes. Bootstrap probabilities from 1000 replicates have been indicated at nodes. The genes showing different topology in DNA-based tree in comparison to protein-based tree have been marked by *. Scale bar represents 0.1 substitutions per nucleotide position. [file 1471-2164-8-242-S3.pdf]
