# Supplementary material for: MADS-box gene family in rice: genome-wide identification, organization and expression profiling during reproductive development and stress
Source: BMC Genomics. 2007 Jul 18;8:242. doi: 10.1186/1471-2164-8-242 (PMC1947970; doi:10.1186/1471-2164-8-242)
Supplement: Additional file 2 — Figure S2. Phylogenetic relationships among rice and Arabidopsis MADS-box proteins. An unrooted tree has been drawn using complete MADS-box proteins of rice and Arabidopsis. Bootstrap values from 1000 replicates have been indicated at nodes. Scale bar represents 0.1 amino acid substitution per site. [file 1471-2164-8-242-S2.pdf]

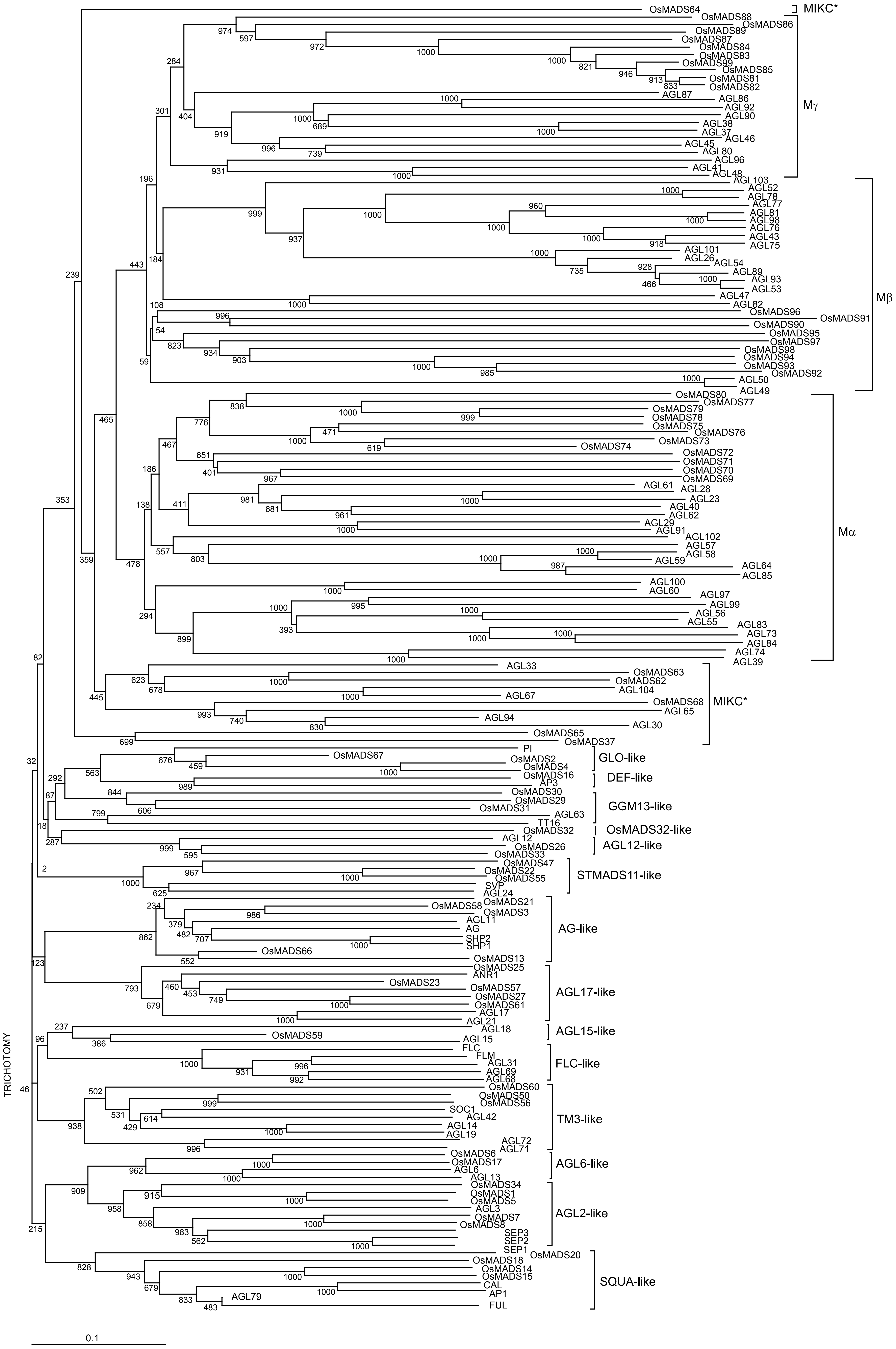
OsMADS64
MIKC*
OsMADS88
OsMADS86
OsMADS89
OsMADS87
OsMADS84
OsMADS83
OsMADS99
OsMADS85
OsMADS81
OsMADS82
AGL87
AGL86
AGL92
AGL90
AGL38
AGL37
AGL46
AGL45
AGL80
AGL96
AGL41
AGL48
Mγ
AGL103
AGL52
AGL78
AGL77
AGL81
AGL98
AGL76
AGL43
AGL75
AGL101
AGL26
AGL54
AGL89
AGL93
AGL53
AGL47
AGL82
OsMADS96
OsMADS91
OsMADS90
OsMADS95
OsMADS97
OsMADS98
OsMADS94
OsMADS93
OsMADS92
AGL50
AGL49
Mβ
OsMADS80
OsMADS77
OsMADS79
OsMADS78
OsMADS75
OsMADS76
OsMADS73
OsMADS74
OsMADS72
OsMADS71
OsMADS70
OsMADS69
AGL61
AGL28
AGL23
AGL40
AGL62
AGL29
AGL91
AGL102
AGL57
AGL58
AGL59
AGL64
AGL85
AGL100
AGL60
AGL97
AGL99
AGL56
AGL55
AGL83
AGL73
AGL84
AGL74
AGL39
Mα
AGL33
OsMADS63
OsMADS62
AGL104
AGL67
OsMADS68
AGL65
AGL94
AGL30
OsMADS65
OsMADS37
MIKC*
PI
OsMADS67
OsMADS2
OsMADS4
OsMADS16
AP3
GLO-like
DEF-like
OsMADS30
OsMADS29
OsMADS31
AGL63
TT16
GGM13-like
OsMADS32
OsMADS32-like
AGL12
OsMADS26
OsMADS33
AGL12-like
OsMADS47
OsMADS22
OsMADS55
SVP
AGL24
STMADS11-like
OsMADS21
OsMADS58
OsMADS3
AGL11
AG
SHP2
SHP1
OsMADS66
OsMADS13
AG-like
OsMADS25
ANR1
OsMADS23
OsMADS57
OsMADS27
OsMADS61
AGL17
AGL21
AGL17-like
AGL18
OsMADS59
AGL15
AGL15-like
FLC
FLM
AGL31
AGL69
AGL68
FLC-like
OsMADS60
OsMADS50
OsMADS56
SOC1
AGL42
AGL14
AGL19
AGL72
AGL71
TM3-like
OsMADS6
OsMADS17
AGL6
AGL13
AGL6-like
OsMADS34
OsMADS1
OsMADS5
AGL3
OsMADS7
OsMADS8
SEP3
SEP2
SEP1
AGL2-like
OsMADS20
OsMADS18
OsMADS14
OsMADS15
CAL
AP1
AGL79
FUL
SQUA-like
TRICHOTOMY
0.1
